# Investigation of the invasion mechanism mediated by the outer membrane protein PagN of *Salmonella* Typhimurium

**DOI:** 10.1186/s12866-021-02187-1

**Published:** 2021-05-21

**Authors:** Emilie Barilleau, Mégane Védrine, Michael Koczerka, Julien Burlaud-Gaillard, Florent Kempf, Olivier Grépinet, Isabelle Virlogeux-Payant, Philippe Velge, Agnès Wiedemann

**Affiliations:** 1 INRAE, Université de Tours, ISP, F-37380 Nouzilly, France; 2 Present Address: Service Biologie Vétérinaire et Santé Animale, Inovalys, Angers, France; 3 Plateforme IBiSA de Microscopie Electronique, Université de Tours et CHRU de Tours, Tours, France; 4 Present Address: IRSD - Institut de Recherche en Santé Digestive, Université́ de Toulouse, INSERM, INRAE, ENVT, UPS, Toulouse, France

**Keywords:** *Salmonella*, Outer membrane protein, PagN, Invasion, Actin, Zipper-like entry pathway

## Abstract

**Background:**

*Salmonella* can invade host cells via a type three secretion system called T3SS-1 and its outer membrane proteins, PagN and Rck. However, the mechanism of PagN-dependent invasion pathway used by *Salmonella enterica*, subspecies *enterica* serovar Typhimurium remains unclear.

**Results:**

Here, we report that PagN is well conserved and widely distributed among the different species and subspecies of *Salmonella*. We showed that PagN of *S.* Typhimurium was sufficient and necessary to enable non-invasive *E. coli* over-expressing PagN and PagN-coated beads to bind to and invade different non-phagocytic cells. According to the literature, PagN is likely to interact with heparan sulfate proteoglycan (HSPG) as PagN-mediated invasion could be inhibited by heparin treatment in a dose-dependent manner. This report shows that this interaction is not sufficient to allow the internalization mechanism. Investigation of the role of β1 integrin as co-receptor showed that mouse embryo fibroblasts genetically deficient in β1 integrin were less permissive to PagN-mediated internalization. Moreover, PagN-mediated internalization was fully inhibited in glycosylation-deficient pgsA-745 cells treated with anti-β1 integrin antibody, supporting the hypothesis that β1 integrin and HSPG cooperate to induce the PagN-mediated internalization mechanism. In addition, use of specific inhibitors and expression of dominant-negative derivatives demonstrated that tyrosine phosphorylation and class I phosphatidylinositol 3-kinase were crucial to trigger PagN-dependent internalization, as for the Rck internalization mechanism. Finally, scanning electron microscopy with infected cells showed microvillus-like extensions characteristic of Zipper-like structure, engulfing PagN-coated beads and *E. coli* expressing PagN, as observed during Rck-mediated internalization.

**Conclusions:**

Our results supply new comprehensions into T3SS-1-independent invasion mechanisms of *S.* Typhimurium and highly indicate that PagN induces a phosphatidylinositol 3-kinase signaling pathway, leading to a Zipper-like entry mechanism as the *Salmonella* outer membrane protein Rck.

## Background

*Salmonella* is a Gram-negative bacterium, belonging to the *Enterobacteriaceae* family. This genus is divided into two species: *S. bongori* and *S. enterica*. The latter consists of six subspecies: *indica*, *diarizonae*, *arizonae*, *salamae*, *houtenae*, and *enterica* [1]. Currently, more than 2600 *Salmonella* serovars have been identified [2]. Warm-blooded animals are mainly infected by strains belonging to *S. enterica* subsp. *enterica* [3]. Depending on the host and the serotype, *Salmonella* leads to a wide variety of diseases ranging from gastroenteritis to systemic typhoid fever in both animals and humans. *Salmonella* is spread by the fecal-oral route and can be transmitted through contaminated water and food. After *Salmonella* ingestion, the bacteria are found in the intestine, where they are able to adhere to the intestinal epithelium and to induce their own entry into host cells. This allows *Salmonella* colonization of the intestinal tract, which constitutes a crucial step in establishing infection [2]. To invade non-phagocytic cells, *Salmonella* expresses several invasion factors: a type III secretion system (T3SS) known as T3SS-1, and two invasins Rck and PagN [4].

For many pathogenic bacteria, T3SS are essential virulence factors composed of several substructures that organize into one needle-like structure called an injectisome. This apparatus serves as an entrance for the bacterial secreted effectors to pass through the inner and outer membranes of the bacterium. When *Salmonella* reaches the small intestine, a neutral pH, a low O_2_ tension, high osmolarity and a high iron concentration induce SPI-1 expression. In contrast, the presence of cationic peptides or bile suppresses its expression. The T3SS-1 allows the injection of bacterial effector proteins directly into the host cell. This promotes massive actin polymerization and ruffles membrane rearrangements, leading to bacterial internalization. This invasion mechanism is described as a Trigger mechanism. The contribution of the T3SS-1 in *Salmonella* pathogenesis has been demonstrated but depends on the host [5].

The outer membrane protein Rck (resistance to complement killing) is encoded by the *rck* open reading frame localized on the virulence plasmid [6]. The transcription of *S.* Typhimurium *rck* gene is regulated by SdiA, a quorum sensing regulator [7], which is activated by acyl homoserine lactones (AHL) produced by other bacteria [8, 9]. The Rck outer membrane protein of *S.* Enteritidis is able to interact with EGFR (epidermal growth factor receptor) expressed on the host cell surface, allowing bacterial invasion [10, 11]. A 46 amino-acid region (from G114 to V159) has been shown to be necessary and sufficient to induce the *S.* Enteritidis invasion mechanism [10]. Between the Rck proteins of *S.* Enteritidis and *S.* Typhimurium, this region is very well preserved except for one amino acid substitution (His to Arg) at position 125. The invasion mechanism induced by Rck of *S.* Enteritidis requires induction of a cellular transduction pathway, which has been well characterized. This includes phosphorylation of tyrosine proteins, and activation of PI 3-kinase (phosphatidylinositol 3-kinase), leading to actin polymerization and weak membrane rearrangement [10, 12, 13]. This invasion mechanism is described as a Zipper mechanism [10]. Rck of *S*. Typhimurium is able to induce the bacterial invasion mechanism [14]. However, the signaling cascade leading to the bacterial internalization has not been characterized. The involvement of Rck-EGFR interaction in *Salmonella* pathogenesis remains unclear. However, a *S.* Typhimurium infection performed in a mouse model of intestinal persistence (an asymptomatic carrier state model) demonstrated that Rck was important for the fitness of *Salmonella* in the intestine [15].

The outer membrane protein, PagN (*phoP*-activated gene), has also been identified as a *Salmonella* invasin [16, 17]. It was first identified in *S.* Typhimurium using a Tn*phoA* random-insertion screening designed to identify PhoP-activated genes [18]. The *pagN* gene is localized on the specific centisome 7 genomic island and is present in most serotypes that have been tested [19–21]. The transcription of *pagN* is regulated by the two-component transcriptional regulatory PhoP/PhoQ system. In response to an acidified environment, low Mg^2+^ concentration or the presence of antimicrobial peptides, PhoQ is auto-phosphorylated and transfers its phosphate to the cytoplasmic DNA-binding protein PhoP that induces or represses the transcription of specific *Salmonella* genes [22]. Lambert et al. were the first to demonstrate that *pagN* deletion in *S.* Typhimurium led to a reduction in *Salmonella* invasion of enterocytes [16, 21]. However, the PagN-mediated invasion mechanism remains poorly characterized at the cellular level. The only information known is that actin polymerization is required for promoting PagN-induced bacterial invasion [17] and that PagN uses extracellular heparan sulfate proteoglycans (HSPG) to invade cells [17]. Concerning the role of PagN in *Salmonella* pathogenesis, several studies have shown that in vivo, a *S.* Typhimurium *pagN* mutant strain (i) induces less pathological signs in the intestine and survives longer compared to its parental strain in streptomycin-treated mice after oral inoculation and (ii) colonizes the spleen of BALB/c/C mice less than the wild-type strain after intra-peritoneal inoculation [21, 23].

In this study, we first took advantage of the large number of *Salmonella* genomes available in Enterobase to revisit the distribution of PagN among the *Salmonella* genus. We investigated the link between HSPG and the PagN-mediated internalization mechanism and then characterized the signaling pathway induced during the PagN invasion mechanism of *S.* Typhimurium within host cells to compare it to the mechanism triggered during the Rck-mediated invasion pathway.

## Results

### PagN invasin is widely distributed and well conserved among the different species and subspecies of *Salmonella*

**Fig. 1:**
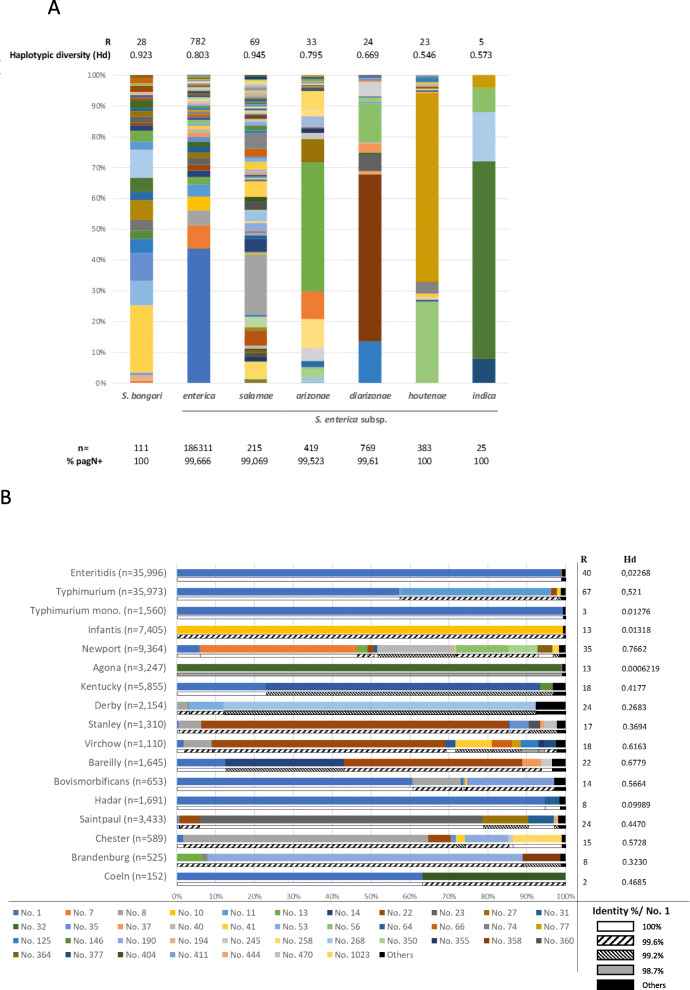
Distribution of allelic variants of *pagN* within *Salmonella* genus. **a** The distribution of *pagN* allelic variants was determined within subspecies *enterica*, *salamae*, *arizonae*, *diarizonae*, *houtenae*, *indica* and species *bongori.* Richness (R) and haplotype diversity (Hd) was measured to evaluate polymorphism. Each colour represents an allelic variant. n represents the number of genomes for each species/subspecies. **b** Distribution of *pagN* allelic variants within 17 out of the 20 most isolated serovars in Europe in 2017. n corresponds to the number of genomes for each serovar. We considered the alleles found in > 1% of the strains in a given serovar. Each allele was designated by the number used in Enterobase. Each colour represents an allelic variant, except black which corresponds to the variants showing frequencies under 0.01 for these serovars. Except for the allelic variants showing frequencies under 0.01, the identity percentages were calculated using the protein encoded by allele designated as No. 1 set as reference

The presence of the *pagN* ORF was previously studied in only a limited number of *Salmonella* strains belonging to the different species, subspecies and serotypes [19–21, 24]. We took advantage of the great number of *Salmonella* genomes available in the extensive Enterobase database to reconsider the distribution of this gene and to study its allele and protein diversity within the *Salmonella* genus (*S. bongori* and *S. enterica*). Consistent with previous works, *pagN* was found at a very high frequency in all *S. enterica* subspecies as well as in *S. bongori* species. The percentage of strains harbouring the *pagN* gene ranged from 99.069% for subspecies *S. enterica* subsp. *salamae* to 100% for *S. bongori* and *S. enterica* subspecies *houtenae* and *indica* (Fig. 1a). A total of 944 allelic variants of the *pagN* ORF were observed, ranging from 700 to 755 nucleotides in length. The allele designated as No. 1 was found to be the most represented within the *Salmonella* genus in the database (42.02% of the recorded genomes). Thus, it was chosen as a reference for all sequence comparisons presented in this section. We then analyzed the distribution of *pagN* allelic variants within *S. bongori* and the six subspecies of *Salmonella enterica* (Fig. 1a). For *S. bongori*, we measured a haplotype diversity index of 0.923, which is relatively high. We found 28 different alleles of the gene, each of them presenting low frequencies (f < 0.1) except the allele designated as No. 51 (f = 0.216). It is interesting to note that only 4 of the 28 allelic variants were shared with the *S. enterica* species. Strains of the non-*enterica* subspecies of *S. enterica* present various haplotype diversity; we found 69, 33, 24, 23 and 5 alleles for *S. enterica* subsp. *salamae*, *S. enterica* subsp. *arizonae, S. enterica* subsp. *diarizonae*, *S. enterica* subsp. *houtenae*, and *S. enterica* subsp. *indica,* respectively. This allelic richness is not related to the number of genomes available for each subspecies. This could explain the variation in the calculated haplotype diversity indexes. However, independently of this diversity, a predominant allele was found in each subspecies; *S. arizonae* was mainly associated with allele No. 110 (f = 0.417), *S. diarizonae* with allele No. 3 (f = 0.538), *S. houtenae* with allele No. 193 (f = 0.613) and *S. indica* with allele No. 87 (f = 0.640). It should be noted that these alleles are specific to these subspecies, as none of these predominant alleles were found in another species or subspecies except allele No. 3, which was found in 3 genomes belonging to subspecies *enterica*. Moreover, the other allelic variants of the gene were rarely shared between non-*enterica Salmonella enterica* subspecies, as only alleles No. 63 and No. 91 were found on two of them (subsp. *arizonae* and *houtenae*).

Finally, for *S. enterica* subsp. *enterica* we measured a relatively high haplotype diversity index of 0.803. Possibly due to the number of available genomes, this subspecies presents the highest number of variants in our sample: 781 alleles are found for this subspecies in the dataset (Fig. 1a) and allele No. 1 is the predominant one (f = 0.424). Other alleles always present low frequencies (f < 0.1) in that sample. Moreover, among the 781 allelic variants, only 17 of them (2.17%) are found in genomes belonging to *S. bongori* species and to other subspecies. Genomes carrying these shared allelic variants belong to a panel of 60 serovars, each showing very different levels of specificity for these variants, their frequencies ranging from 0.0001 to 1. Amino-acid sequences of the predominant alleles of *S. bongori* and non-*enterica* subspecies of *S. enterica* were compared to the translated sequence of allele No. 1. They all have a very high percentage of identity with our reference allele (ranging from 82.8 to 95.0%), thus highlighting a high conservation of the PagN protein in the *Salmonella* genus (data not shown).

We next investigated the distribution of *pagN* allelic variants among the 20 most frequently isolated *Salmonella enterica* subsp. *enterica* serovars in humans in Europe in 2017 [25]. It should be noted that genomes of three of these serovars, i.e. serovars Naples, Java and Kottbus, were not available in Enterobase when the genomes were retrieved for our study. This represented 112,662 genomes, in which 268 alleles were identified. Among these allelic variants, 240 were serovar-specific. Given the low distribution of some allelic variants in this dataset, we only considered the allelic variants carried by at least 1% of the strains in at least one of these 17 serovars for subsequent analysis. This represented a total of 40 alleles of the *pagN* gene (covering 98.53% of the 112,662 selected genomes). Under this scheme, we observed that 11 out of the 17 serovars were predominantly associated with one allele. For example, allele designated as No. 1 was found at very high frequencies in genomes of *S.* Enteritidis (f = 0.988) and of the monophasic variant of *S.* Typhimurium (antigenic formula: 4;[5];12:i:-) (f = 0.993) strains, as well as allele No. 10 that was found at similar frequencies in *S.* Infantis genomes (f = 0.993). On the other hand, we also observed other serovars associated with a larger range of alleles, such as *S.* Newport, *S.* Virchow or *S.* Bareilly, consequently showing higher haplotype diversity indexes (Fig. 1b). The amino acid sequence alignment of these 40 allelic variants showed high identity with allele No. 1, ranging from 98.7 to 100% (Fig. 1b).

Taken together, these results confirm that *pagN* is widely distributed within the *Salmonella* genus, and demonstrate that the encoded protein is well conserved among species, subspecies and serovars. They also highlight some allelic specificity at the species, subspecies and serovar levels. This high conservation of PagN suggests an ubiquitous role of this protein, independent of the strain serovar-specificity, and of the pathogenic potential of the strains toward their hosts, although we cannot exclude that some substitutions could be responsible for these phenotypes.

### PagN-mediated invasion mechanism depends on the host cell line

**Fig. 2:**
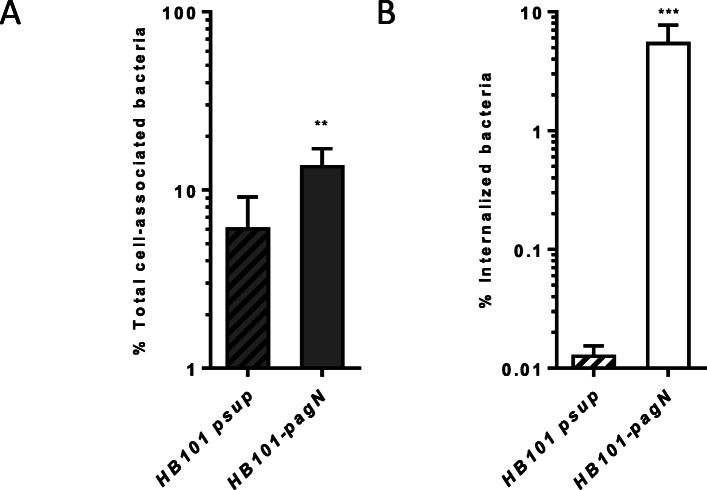
PagN is able to induce both adhesion and invasion depending on the cell line. CHO cells were infected with *HB101-psup* (hatched bars) or *HB101-pagN* strain (empty bars) at 37 °C for 1 h (MOI 1:10). The percentages of total cell-associated (**a**) and internalized (**b**) bacteria have been calculated as described in Materials and Methods. Data show mean values ± SD acquired from three independent experiments with two infected wells per experiment. Data were compared using a Mann Whitney test (***p < 0.001, **p < 0.01)

PagN of *S.* Typhimurium has previously been shown to mediate both adhesion to and invasion of CHO cells [16]. Prior to characterizing the invasion mechanism mediated by PagN, we first decided to confirm these results. PagN of *S.* Typhimurium was chosen and a non-invasive *E. coli* HB101 strain harboring either pSUP202 (*HB101-psup*) or pSUP202-PagN (*HB101-pagN*) was used as standard in vitro culture conditions are not suitable for PagN production by *S.* Typhimurium [22]; Holbert et al. unpublished]. The percentage of total cell-associated and internalized bacteria was determined using standard adhesion and invasion assays. As shown in Fig. 2, we observed that the percentage of total cell-associated and internalized *HB101-pagN* strain was increased 2- and 400-fold, respectively compared to the control strain (*HB101-psup*). Our results confirm that PagN is able to induce bacterial adhesion to and invasion of epithelial cells.

**Fig. 3:**
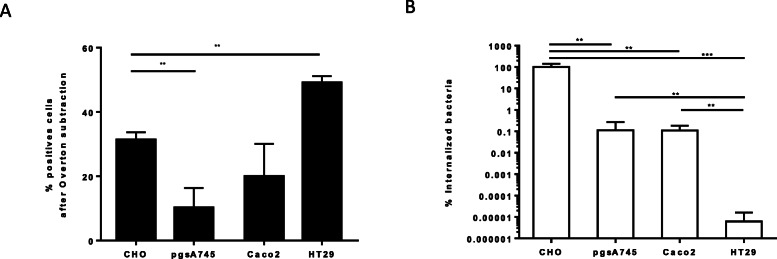
The presence of heparan sulfate is not correlated to the level of PagN-mediated internalization. **a** The distribution of HS was analyzed on the cell surface of CHO, pgsA745, Caco-2 and HT29 cell lines by flow cytometry using a specific anti-HS antibody. The percentage of HS positive (HS+) cells was calculated using histogram subtraction (method of Overton) for each cell line as described in the Materials and Methods. **b** CHO, pgsA745 and Caco-2 cells were infected with *HB101-pagN* strain at 37 °C for 1 h (MOI 1:10). The percentage of internalized bacteria was calculated as described in Materials and Methods and related to values obtained for CHO cells, set at 100%. Results represent mean values ± SD obtained from three independent experiments. Results were compared using a Mann Whitney test (***p < 0.001, **p < 0.01)

Lambert and Smith showed that PagN utilizes HSPG to invade mammalian cells [17]. HSPG are membrane-anchored proteins with covalently attached glycosaminoglycan side-chains consisting of heparan sulfate (HS) [26]. In order to characterize better the role of HSPG in PagN-mediated invasion, we first determined the expression of HS on the cell surface of different cell lines by flow cytometry using a specific monoclonal anti-HS antibody. The different cell lines chosen were: (i) Caco2 cells, which are mainly used to study the intestinal invasion of *Salmonella* [27]; (ii) HT29 and CHO cell lines as they were previously used to study PagN-mediated invasion mechanism [16, 21]; (iii) proteoglycan-deficient CHO cell line (pgsA745 cells) as a control. The mean percentage of HS positive (HS+) cells showed that HS were detectable on the surface of each cell line but at different levels. As shown in Fig. 3a, the mean percentage of HS+ cells is similar in CHO and Caco2 cells, while it is significantly lower in pgsA745 cells and higher in HT29 cells. Next, the ability of *HB101-pagN* to invade these different cell lines was measured. As expected, we observed that the mean percentage of internalized *HB101*-*pagN* was 1000-fold lower in pgsA745 than CHO cells, confirming the results of Lambert et al [17]. Surprisingly, the percentage of internalized bacteria was identical in Caco2 and pgsA745 cells and 10,000-fold lower in HT29 cells than in pgsA745 cells (Fig. 3b). These results demonstrated that the invasion ability of an *E. coli* strain expressing PagN is not related to the HS exposed on the host cell surface.

Taken together, these data demonstrate that PagN is able to mediate cell invasion but regardless of the HS, suggesting that HSPG are involved but not sufficient to allow the invasion mechanism mediated by PagN-mediated invasion.

### PagN-mediated internalization requires β1 integrin

**Fig. 4:**
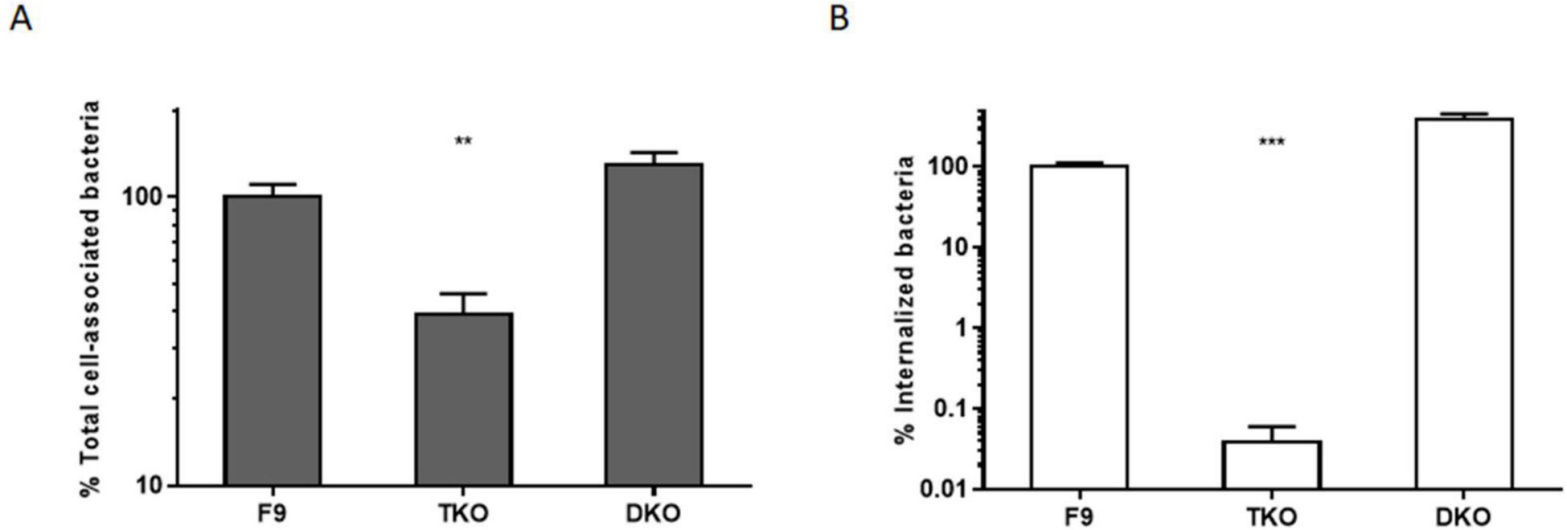
The binding and invasiveness of *Yersinia* Invasin require β1 integrin receptor. F9, triple knockout (TKO) and double knockout (DKO) cells were infected with *MC-InvGFP* strain at MOI 1:10 at 37 °C for 1 h. The percentage of total cell-associated (**a**) and intracellular (**b**) bacteria was determined as described in Materials and Methods. Obtained results are expressed relative to values obtained with F9 cells, set at 100%. Results were compared using a Mann Whitney test (***p < 0.001, **p < 0.01)

**Fig. 5:**
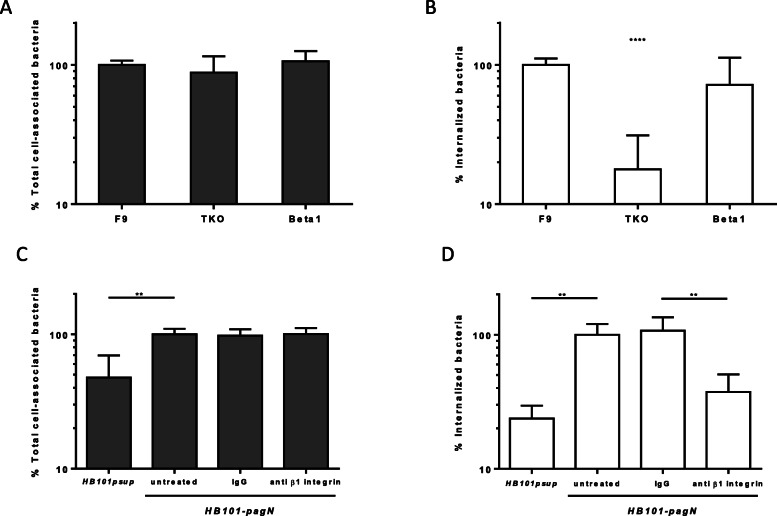
HSPG and β1 integrin cooperate to induce PagN-mediated internalization. **a-b** Parental F9, triple knockout (TKO) and double knockout (DKO) cells were infected with *HB101-pagN* at MOI 1:10 at 37 °C for 1 h. **a** Percentages of total cell-associated bacteria and (**b**) internalized bacteria were determined as described in the Materials and Methods. Results shown are expressed relative to values got with the parental F9 cells (F9), set at 100%. **c-d** pgsA745 cells were untreated or treated with integrin β1 blocking antibody and IgG at 50 μg/mL for 30 min at 4 °C prior to the addition of *HB101-pagN* or *HB101-psup* at MOI 1:10 for 1 h at 37 °C. **c** Percentages of total cell-associated bacteria and (**d**) internalized bacteria were determined as described in the Materials and Methods. Results obtained are expressed relative to values obtained for untreated cells infected with *HB101-pagN*, arbitrarily set at 100%. Results represent means ± SD of three independent experiments with two infected wells per experiment. Results were compared using a Mann Whitney test (****p < 0.0001, **p < 0.01)

According to the literature, HSPG may act as co-receptors for downstream cellular signaling events triggered by integrins [28, 29]. To assess the role of β1 integrin in PagN-mediated invasion, cells that were deficient for β1 chain integrin production were used. F9 cells carry three copies of the gene encoding the β1 integrin chain, and TKO (triple knockout) cells fail to express β1 integrin chain due to insertions in each of the three copies. DKO (double knockout) cells retain one intact copy of the *β1 integrin* gene and thus retain production of β1 integrins [30]. Targeted deletion of β1 integrins in F9 cells affects morphological differentiation but not tissue-specific gene expression. As control, an *E. coli* MC1061 strain which overexpresses the *Yersinia enterocolitica* Invasin protein (*MC-InvGFP*) allowing binding to β1 integrin receptor and subsequent invasion into mammalian cells was used [31]. The ability of *HB101-pagN* and *MC-InvGFP* to bind to and invade F9, TKO and DKO cells was thus compared. As expected, Invasin-expressing strain was able to adhere and invade more efficiently cells expressing β1 integrin receptor (Fig. 4). As shown in Fig. 5b, the percentage of internalized *HB101-pagN* was significantly higher in F9, and DKO cells, expressing β1 integrin than in TKO cells, Indeed, an 8- and 5-fold decrease in invasion was observed in TKO cells compared to the other two cell lines, respectively. In contrast, the absence of β1 integrin resulted in similar level of the total number of cell-associated bacteria (Fig. 5a). This provides evidence that PagN-mediated internalization but not adhesion depends on β1 integrin.

The involvement of β1 integrin in PagN-mediated internalization led us to investigate the cooperation of HSPG and β1 integrin in this process. To this end, the ability of *HB101-pagN* to invade pgsA745 cells pre-treated with a blocking anti-β1-chain integrin antibody or with IgG as a control was measured. As shown in Fig. 5d, the pre-treatment with an anti-β1 integrin antibody significantly reduced the percentage of internalized *HB101-pagN* to a level similar to that obtained with the *HB101-psup* strain. In addition, the number of internalized *HB101-pagN* obtained in cells untreated or treated with IgG was similar. The difference observed is not due to a difference in the ability of the bacteria to adhere to cells as the pre-treatment with either an anti-β1-chain integrin antibody or IgG resulted in similar level of the total number of cell-associated bacteria (Fig. 5c). These data confirm that the internalization triggered by PagN depends on HSPG and β1 integrin.

As CHO cells allowed a high level of PagN-mediated invasion, the following experiments aimed to characterize PagN-mediated internalization were performed only with this cell line.

### *S.* Typhimurium Rck- and PagN- mediated internalization hijacks the host cellular actin, leading to a zipper mechanism

**Fig. 6:**
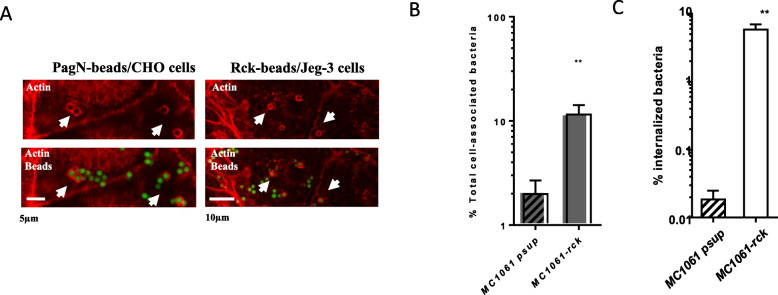
PagN and Rck of *S.* Typhimurium are able to bind to and induce bacterial invasion, leading to a local remodeling of the host actin cytoskeleton. **a** CHO and Jeg-3 cells were incubated with either PagN- or Rck-coated beads, respectively. After 30 min of contact between cells and coated beads at 37 °C, cells were washed and then stained by immunofluorescence. Horizontal sections of cells obtained with confocal laser scanning microscopy shows actin staining in red and overlay of beads in green and actin. Representative images are shown with an arrow, indicating the site of actin polymerization and typical structural morphologies. **b**-**c** Jeg-3 cells were infected with *MC1061-psup* or *MC1061-rck* strain for 1 h at 37 °C (MOI 1:10). The percentages of total cell-associated (**b**) and internalized (**c**) bacteria were determined as described in the Materials and Methods. Results are mean values ± SD acquired with three independent experiments with two infected wells per experiment. Results were compared using a Mann Whitney test (**p < 0.01)

To demonstrate that PagN alone can induce cell adhesion, actin cytoskeletal rearrangement and cell invasion, a model with 2 μm latex beads coated with PagN fused to Glutathione S-Transferase (PagN-beads) has been established and GST-coated beads were used as control. GST-PagN fusion protein was produced and purified from BL21 pLysS harboring pGEX4T2-PagN. Adhesion of PagN- and GST-coated beads to CHO cells was detected by their green autofluorescence and internalization of those beads by their double fluorescence due to labelled antibodies against GST and green autofluorescence (Table 1). Actin recruitment at the entry site was visualized using confocal microscopy. F-actin was stained with phalloidin conjugated to rhodamine (Fig. 6a) and coated beads were in green due to their green autofluorescence. Confocal images were generated and showed a local actin polymerization underneath PagN-beads (Fig. 6a). As expected, GST-coated beads were rarely found associated with cells as previously observed by Rosselin et al. (Table 1). As shown in Fig. 6a and Table 1, PagN is able to mediate adhesion, actin rearrangement and invasion into CHO cells. These data show that PagN of *S.* Typhimurium induces adhesion and actin rearrangement, leading to bacterial invasion.

**Table 1:** Abilities of latex beads coated with different GST fusion proteins to bind and mediate actin recruitment and internalization

Coated beads	Adhesion	Actin recruitment	Internalization
GST	–	–	–
GST-113-159Rck	+++	+++	+++
GST-PagN	+++	+++	+++

(−): non-detectable; (+) low, (++) medium and (+++) high level

*S.* Typhimurium expresses two outer membrane proteins, PagN and Rck. Both induce actin polymerization, leading to bacterial internalization [10, 16]. To compare the PagN-mediated invasion process with the mechanism induced by Rck of *S.* Typhimurium, we performed several experiments to confirm that Rck of *S.* Typhimurium had the same properties as Rck of *S.* Enteritidis [10, 12, 13]. Two models were used: (i) a non-invasive *E. coli* strain, which overexpressed Rck of *S.* Typhimurium (*MC1061*-*rck)* and its control *E. coli* strain, only harboring pSUP202 (*MC1061-psup*) and (ii) beads coated with the 114–159 peptide of Rck fused to Glutathione S-Transferase (Rck-beads) and its control GST-beads. The peptide 114–159 of *S.* Enteritidis Rck has been shown to be sufficient and necessary to induce adhesion, actin polymerization and internalization [10]. First, the adhesion and invasion level of *MC1061-rck* and *MC1061-psup* were compared in Jeg3 cells, a cell line already shown to be permissive to Rck-mediated adhesion and invasion [10, 12]. As shown in Fig. 6b-c, *MC1061*-*rck* strain adhered to and invaded Jeg3 cells about 6 and 300 times more efficiently, respectively, than the control *MC1061-psup* strain did. Then, we confirmed that Rck-beads could induce cell adhesion, actin cytoskeletal rearrangement and cell invasion. As observed in Table 1 and Fig. 6a, Rck-beads are able to adhere and to induce host actin rearrangement or particle internalization. Taken together, our results show that bacteria expressing Rck, as well as beads coated with Rck are good models to characterize the internalization mechanism induced by Rck of *S.* Typhimurium and to compare it to the PagN-mediated invasion mechanism.

**Fig. 7:**
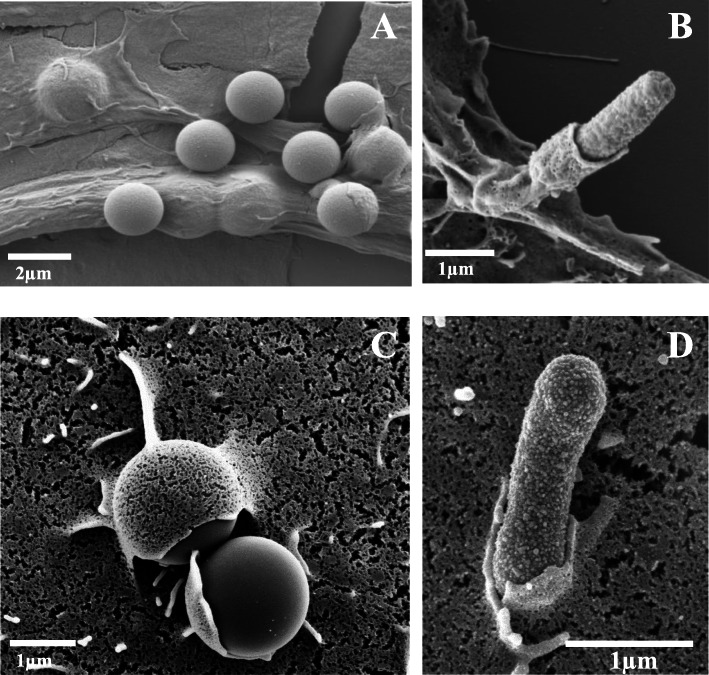
Rck and PagN of *S.* Typhimurium mediate a Zipper-like entry mechanism. **a-b** CHO cells were incubated with PagN-beads (**a**) or *HB101-pagN* (**b**). **c-d** Jeg-3 cells were incubated with Rck-beads (**c**) or *MC1061-rck* (**d**). After 1 h, the cells were washed and then processed for scanning electron microscopy

The interaction of *HB101*–*pagN* or the PagN-beads with CHO cell surface were further analyzed by scanning electron microscopy and compared to the membrane rearrangement observed with *MC1061-Rck* or Rck-beads incubated with Jeg3 cells. In Fig. 7, the different stages of PagN- (Fig. 7a-b) and Rck- (Fig. 7c-d) mediated invasion can be pictured, i.e. adherent bacteria or beads associated with cellular extension membrane, partially engulfed bacteria or beads with a membrane rearrangement and totally internalized beads. The PagN-dependent membrane rearrangements are weak and similar to the membrane engulfment observed during the Rck-mediated invasion. This suggests that PagN mediates a Zipper-like entry mechanism like the outer membrane protein Rck.

### The signaling pathway induced by *S.* Typhimurium Rck- and PagN-mediated internalization, involves the PI 3-kinase pathway

**Fig. 8:**
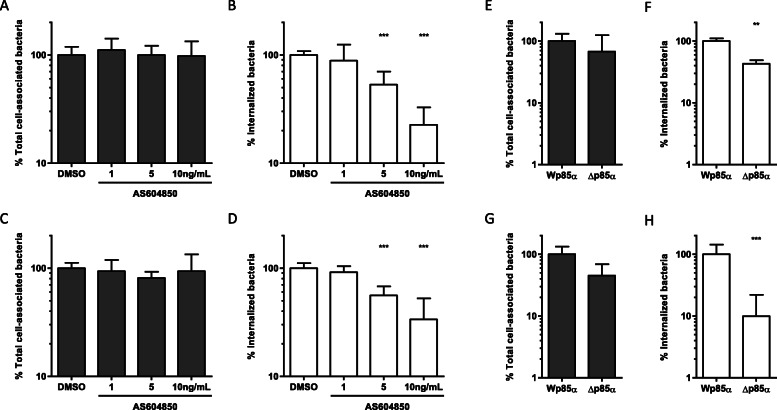
Class I PI 3-kinase p85α-p110 is required for Rck- and PagN-mediated internalization. CHO (**a-b**) or Jeg-3 (**c-d**) cells were incubated with AS604850 at the indicated concentrations for 2 h 30 prior to the addition of *HB101-pagN* (**a-b**) or *MC1061-rck* (**c-d**) at MOI 1:10 for 1 h at 37 °C. Percentages of total cell-associated bacteria (**a-c**: grey bars) and internalized bacteria (**b-d**: white bars) were determined as described in the Materials and Methods. Results acquired with drugs are expressed relative to values acquired for the same amount of DMSO-containing medium (DMSO), set at 100%. CHO (**e-f**) and Jeg-3 (**g-h**) cells transfected with ∆p85α and Wp85α were infected with *HB101-pagN* (**e-f**) or *MC1061-rck* (**g-h**) at MOI 1:10 at 37 °C for 1 h. The percentages of total cell-associated bacteria (**e-g**: grey bars) and internalized bacteria (**f-h**: white bars) were calculated and expressed relative to values obtained for Wp85 α transfected cells, set at 100%. Values represent means ± SD of three independent experiments with two infected wells per experiment. Results were compared using a Mann Whitney test (***p < 0.001, **p < 0.01)

The β1 integrin and Rck of *S.* Enteritidis trigger a signaling cascade involving the class I PI 3-kinase p85α-p110 heterodimer pathway [12, 32]. To investigate the specificity of this signaling cascade with regard to the mechanism induced by PagN and Rck of *S.* Typhimurium, the effect of the p110 heterodimer inhibitor (AS-604850) on adhesion and entry of *HB101–pagN* and *MC1061-rck* was examined. Addition of AS-604850 to CHO and Jeg3 cell monolayers before adherence and invasion assays had no effect on adhesion as the number of associated *HB101*-*pagN* and *MC1061-rck* bacteria were similar that of DMSO-treated cells (Fig. 8a-c). However, the number of internalized Rck-expressing bacteria decreased in a dose-dependent manner with this inhibitor. Similar results were observed with PagN-expressing bacteria (Fig. 8b-d).

To obtain clear evidence that the class I PI 3-kinase p85α-p110 is needed for the invasion mechanism induced by PagN and Rck of *S.* Typhimurium, the dominant negative form of p85α (Δp85α) and the wild-type form of p85α (Wp85α) were stably overexpressed in CHO and Jeg3 cells. Thirty-five amino acids from residues 479–513 of p85α are deleted in the dominant negative form, known to inhibit PI 3-kinase activation [12, 33]. The ability of *HB101-pagN* and *MC1061-rck* to bind to and invade these stably transfected cells was thus compared. As shown in Fig. 8e-h, the number of internalized bacteria expressing either PagN or Rck was significantly lower in ∆p85α cells, compared to that in Wp85α cells, while no significant change was highlighted in the number of cell-associated bacteria between the transfected cell lines. These results indicate that the p85α-p110 heterodimer plays a role in the signaling pathway induced by both PagN and Rck, leading to bacterial internalization into cultured cells.

**Fig. 9:**
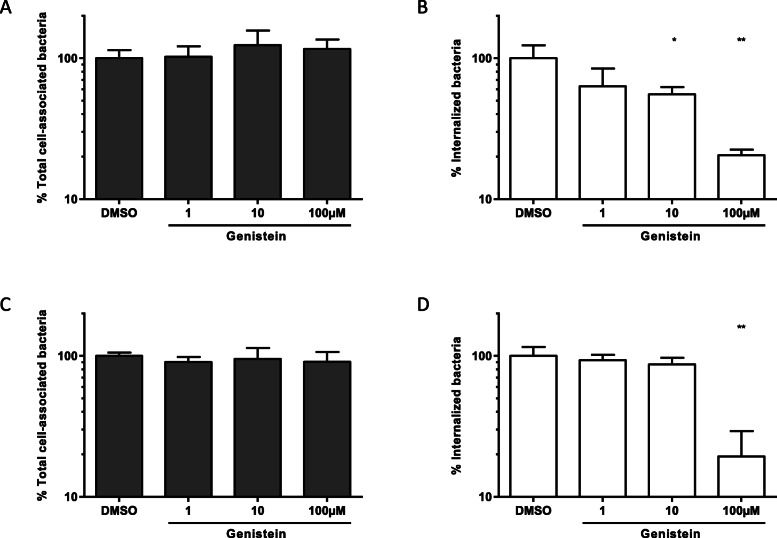
Protein tyrosine kinases are required for Rck- and PagN-mediated internalization. CHO (**a-b**) or Jeg-3 (**c-d**) cells were incubated with genistein at the indicated concentrations for 15 min prior to the addition of *HB101-pagN* (**a-b**) or *MC1061-rck* (**c-d**) at MOI 1:10 for 1 h at 37 °C. Percentages of total cell-associated bacteria (**a-c**: grey bars) and internalized bacteria (**b-d**: white bars) were determined as described in the Materials and Methods. Results obtained with drugs are expressed relative to values obtained for the same amount of DMSO-containing medium (DMSO), arbitrarily set at 100%. Data were compared using a Mann Whitney test **p < 0.01, *p < 0.05)

Activation of the PI 3-kinase requires the interaction of the SH2 domains of the p85 subunit with tyrosine phosphorylated proteins [34]. To assess the role of protein tyrosine kinases in *S.* Typhimurium PagN- or Rck- mediated internalization, the effect of treatment with genistein, a specific inhibitor of protein tyrosine kinases, was analyzed on PagN or Rck-mediated adhesion and internalization. As shown in Fig. 9, the PagN- or Rck-mediated invasion decreased in the presence of genistein in a dose-dependent manner, whereas no effect on PagN- or Rck-mediated adhesion was highlighted. These results show that the PagN- or Rck-dependent internalization mechanism requires tyrosine phosphorylation.

Taken together, these data demonstrate that like for Rck invasion, PagN of *S.* Typhimurium induces and requires the PI 3-kinase signaling pathway to trigger bacterial internalization.

## Discussion

*S.* Typhimurium takes advantage of different strategies to invade host cells. The major determinant of this invasiveness described in the literature is the T3SS-1, but other T3SS-independent mechanisms are also used by *S.* Typhimurium to gain entry into host cells such as the outer membrane proteins, Rck and PagN. Our study aimed to characterize better PagN and the entry pathway induced by this outer membrane protein. Previously, the presence of the *pagN* ORF was studied in few strains or genomes and this ORF was shown to be present in all the strains tested [19, 21, 24]. Based on Enterobase, we were able to confirm the presence of the *pagN* ORF on a very large dataset of more than 188,000 genomes of *Salmonella,* including genomes of the two *Salmonella* species, i.e. *S. bongori* and *S. enterica,* of all *S. enterica* subspecies and of 465 different serovars of *S. enterica* subsp. *enterica*. More than 99.6% of the tested genomes were positive for the gene, confirming studies based on a more limited number of strains/genomes. Moreover, for the first time, we highlighted some allelic specificity at the species, subspecies and serovar levels. Despite this allelic specificity, allelic variants show a very high conservation within *S. enterica* subsp. *enterica*, and also within other subspecies and species but to a lower extent. This high conservation does not, however, predict the functionality of the protein as it has recently been shown that only one substitution in loop 1 or 2 could be sufficient to increase the adhesive and invasive properties of PagN [35]. Further studies are required to decipher the amino acids/peptides important for PagN function, especially those in the predicted outer membrane loops.

Based on several lines of evidence, Lambert and Smith indicated that the epithelial cell surface receptor of PagN may be a HSPG [17]. They showed that *S.* Typhimurium and recombinant *E. coli* expressing PagN had a significant decrease in the ability to invade cells presenting under-glycosylated proteoglycans and that PagN-mediated internalization was significantly reduced in cells pre-treated with exogenous glycosaminoglycans and heparin. In our study, we compared the level of PagN-mediated invasion in different cell lines and identified them as permissive, resistant, and intermediate cells to this invasion process. However, the quantification of HS, which occurs as HSPG, on the surface of cells of these lines did not allow us to establish a link with the permeability/resistance of the cells to PagN-mediated invasion. Indeed, HT29 cells, which are extremely resistant to PagN-mediated invasion, express the highest amount of HS on their surface compared to the other cell lines tested. Therefore, the susceptibility/resistance to the PagN-mediated invasion is not proportional to the distribution of HS on these cell lines. As the PagN-mediated invasion is reduced in HSPG deficient cells compared to parental cells, all these results suggest that HSPG are necessary but not sufficient for *S.* Typhimurium internalization and thus could be considered as a co-receptor in PagN-mediated invasion.

The literature describes clearly that HSPG can be conjugated onto a variety of proteins to induce a signaling pathway, allowing the invagination of the cell membrane. Exosomes, cell penetrating peptides, viruses, bacteria, growth factors, lipoproteins and morphogens among other ligands penetrate cells through HSPG-mediated endocytosis. This leads to the modulation of biological activities of these molecules by influencing the duration and potency of the signaling. HSPG may thus act as a co-receptor for different cell surface receptors. The ligand-binding to HSPG results in conformational change of ligand, allowing it to present to endocytosis receptors with a high-affinity [36]. HSPG endocytosis seems thus not to be limited to one particular pathway, and changes depending on the type of extracellular ligand and cellular context. HSPG are exclusively produced by epithelial cells [37] and they have been detected in the intestine of humans [38] and mice [39]. Binding to host cells through recognition of HSPG has been associated with the invasion of several bacteria such as *Neisseria gonorrhoeae* [40]. Syndecan-1 and -4 are involved in attachment of host cells by *Neisseria gonorrhoeae* [41, 42], acting as co-receptor to facilitate bacterial internalization thanks to the β1 integrin receptor [43]. Syndecan-1 is a highly conserved, multifunctional receptor and the major HSPG expressed on intestinal epithelial cell surfaces [44]. As Syndecan-1 and β1 integrin generate a signal via the PI 3-kinase pathway [45] and undergo endocytosis upon clustering [46], our results reinforce the observation of Lambert and Smith [17], suggesting that Syndecan-1 is involved in the PagN-mediated internalization mechanism.

Signaling molecules are differentially targeted by bacteria to promote invasion. During Zipper bacterial invasion, the receptor-ligand interaction leads to a PI 3-kinase signaling pathway and the stimulation of actin cytoskeletal rearrangements, promoting the advance of pseudopods [47]. In this study, the signaling pathway induced by *S.* Typhimurium Rck and PagN, leading to bacterial internalization was characterized and compared to elucidate when the bacteria use these two outer membrane proteins. Using a pharmacological inhibitor and a dominant negative mutant of class I PI 3-kinase, we demonstrated in this study that the signaling transduction induced by *S.* Typhimurium Rck requires (p85-p110) PI 3-kinase as Rck of *S.* Enteritidis. In addition, the use of these tools also allowed us to show that (p85-p110) PI 3-kinase is required for the signaling transduction which leads to PagN-mediated invasion without affecting attachment.

As protein tyrosine kinase is an upstream signaling molecule of (p85-p110) PI 3-kinase during the Rck-mediated internalization of *S.* Enteritidis, the involvement of protein tyrosine kinase in PagN- and Rck-mediated invasion of *S.* Typhimurium was investigated. Our data highlighted that phosphorylation of tyrosine is required for the Rck- and PagN-mediated invasion by showing that the invasion level induced by Rck or PagN was significantly reduced in cells treated with the inhibitor genistein. Our data demonstrate that the signaling induced by Rck- and PagN-mediated entry of *S.* Typhimurium has similarities and involves the PI 3-kinase pathway, to allow bacterial internalization.

The scanning electron microscopy analysis of the interaction between either *HB101-pagN* or PagN-beads and epithelial host cell surface revealed a Zipper-like structure surrounding the adherent bacteria and coated beads. These data, combined with the fact that (i) β1 integrin is required for PagN-mediated internalization and has been described in the literature as a receptor, allowing Zipper-like process and (ii) PagN alone mediates a PI 3-kinase signaling pathway, leading to internalization, strongly suggest that PagN and Rck of *S.* Typhimurium trigger cell invasion through a Zipper-like mechanism, as during Rck-mediated internalization of *S.* Enteritidis [10].

The transcription of *pagN* is directly regulated by PhoP/PhoQ [48]. This two-component system is activated by an acidified environment and a low Mg^2+^ concentration, conditions found intracellularly, inside the *Salmonella*-containing vacuole (SCV). By contrast, this environmental condition is not favorable for Rck production (data not shown). Currently, Rck production is known to be directly regulated by SdiA in an AHL–dependent manner [8, 49] despite the fact that some studies have shown that *Salmonella* does not produce AHLs and that some evidence suggests a lack of AHL signaling molecules in the mammalian intestine [15, 50]. In addition, the genes encoding T3SS-1 are induced extracellularly (by high osmolarity and low oxygen concentration) and downregulated after internalization [51, 52]. Altogether, these data show that *S.* Typhimurium expresses and uses its invasion factors under different environmental conditions, suggesting a specificity of the entry route by *Salmonella* strains which depends on the host cell environment.

In *Salmonella* pathogenicity, the importance of Rck- and PagN-mediated invasion remains unknown. In vivo studies in mice suggest an intestinal role of PagN and Rck [15, 21, 23] and lead to several hypotheses. In the intestine, EGFR, HSPG and β1 integrins are present on the surface of epithelial cells constituting the intestinal crypt base [53]. As *Salmonella* can target intestinal stem cells [54], Rck- and PagN- mediated invasion could occur at the lumen site of the crypts. In addition, M cells express β1 integrins on their luminal side [55]. *Salmonella* is able to invade and destroy M cells, leading to invasion and colonization of the intestine [56]. As a *Salmonella* strain with a nonfunctional T3SS-1 is still able to invade M cells in mouse intestine, it is possible that the PagN-mediated invasion may be targeting M cells [57–59]. Moreover, EGFR, HSPG and β1 integrins are found to be expressed on the basolateral membrane of villus enterocytes [60–62]. As *Salmonella* can cross the intestinal barrier and exit on the basolateral side [63, 64], one hypothesis could be that Rck and PagN allow *Salmonella* invasion of enterocytes via the basolateral side. Based on the fact that *Salmonella* uses its T3SS-1 to alter epithelial cell polarity to allow bacterial invasion, another possibility thus could be that initial *Salmonella* invasion on the apical side induces a redistribution of HSPG, β1 integrins and EGFR on the cell surface, allowing PagN- and Rck- mediated invasion from the apical side of epithelium [65].

*Salmonella* colonization is not limited to the intestinal tract. Indeed, *Salmonella* can disseminate and colonize systemic sites [66]. Considering that (i) a *S.* Typhimurium strain with a non fonctional T3SS-1 is still able to infect mice and colonize systemic organs such as the liver [67], (ii) *Salmonella* colonization of mice liver is significantly reduced in absence of PagN [21, 23], (iii) EGFR and β1 integrins are expressed on the cell surface of hepatocytes [68, 69], another hypothesis could be that PagN- and Rck-mediated invasion allow the bacterial colonization of systemic organs such as the liver. The fact that Rck confers resistance to complement-mediated killing, reinforces this hypothesis [70]. The next step now is to investigate these different hypotheses using organoid models as a primary intestinal epithelium in vitro culture model.

## Conclusions

Overall, the comparison of PagN- and Rck- mediated invasion of *S.* Typhimurium highly indicates that PagN induces a phosphatidylinositol 3-kinase signaling pathway, leading to a Zipper-like entry mechanism as the *Salmonella* outer membrane protein Rck. The investigation of the molecular elements of the signal transduction mediated by PagN supplies new comprehensions into T3SS-1-independent invasion mechanisms and could help to explain the specificity of each internalization process pathway.

## Methods

### Bioinformatics analyses

We retrieved the wgMLST profiles of 195,555 *Salmonella* strains recorded in Enterobase, an online platform that assembles draft genomes from Illumina short reads [71] on March 27, 2019. Among these strains, we kept only those presenting consistent serovar predictions (obtained using the online typing tool SISTR [72]). Consequently, the analysis was performed on 188,233 genomes. Allelic data at the *pagN* locus (referred to as STMMW_03171 in Enterobase’s wgMLST scheme) were retrieved from the dataset and the distribution of the alleles was studied according to the *Salmonella* species, subspecies and serovars. The diversity among nucleotide sequences was calculated at each taxonomic level (species, subspecies and serovar) using Nei’s haplotypic diversity (Hd), computed using the R-package pegas [73, 74]. After translation, sequences were aligned with the protein encoded by allele designated as No. 1 in Enterobase using ClustalW implemented in the software Geneious 10.2.2 (https://www.geneious.com).

### Cell lines and reagents

Various mammalian cell lines were used in this study. Parental F9, and integrin β1 double (DKO) and triple (TKO) knockout F9 embryonal carcinoma cell lines (kindly provided by Dr. C. Le Bouguenec, Institut Pasteur Paris, France) as well as Chinese Hamster Ovary (CHO) cells (ATCC: CCL-61) and HT29 cells, human caucasian colon adenocarcinoma cells (ATCC: HTB-38) were cultured in DMEM (Dulbecco’s modified Eagle’s medium, Gibco) containing glucose 25 mM supplemented with FBS 10% (fetal bovine serum; Sigma), L-glutamine 2 mM (Gibco) in a humidified atmosphere at 37 °C and CO_2_ 5%. Jeg-3 cells, human epithelial placental cells (ATCC: HTB-36), and the stably transfected cells, Jeg-3 Wp85α and ∆p85α, were grown in MEM medium containing Glutamax (Gibco), FBS 10%, non-essential amino acids 1 mM and sodium pyruvate 1 mM (Gibco) [12]. Caco-2 cells (ATCC: HTB-37) are human colonic epithelial cell lines cultured in DMEM supplemented with FBS 20%, nonessential amino acids 1 mM, sodium pyruvate 1 mM and L-glutamine 2 mM. pgsA745 cells (ATCC: CRL-2242) referred to as ∆*XylT* [75] were routinely cultured in F-12 K medium (Kaighn’s Modification of Ham’s F-12 medium; ATCC) supplemented with FBS 10%.

All inhibitors were dissolved in DMSO (dimethyl sulfoxide, Sigma) at the following stock concentration: AS604850 (Sigma at 35 mM); Genistein (Calbiochem at 100 mM). In drug-treated cells, the maximum final concentration of DMSO never exceeded 0.1% (v/v).

### Bacterial strains and growth conditions

In Table 2, the bacterial strains used in this study are listed. Bacteria were routinely cultured in LB (Luria-Bertani) broth overnight with shaking at 150 rpm at 37 °C with the corresponding antibiotic: tetracyclin (Tc, Sigma) 12.5 μg/ml, chloramphenicol (Cm, Sigma) 34 μg/ml and carbenicillin (Cb, Sigma) 100 μg/ml.

**Table 2:** Bacterial strains and plasmids used in this study

Strain or Plasmid	Relevant characteristeristics	Source or reference
**Strains**		
HB101	Noninvasive laboratory strain (s*upE44 hsdS20*(r_B_^−^m_B_^−^) *recA13 ara-14 proA2 lacY1 galK2 rpsL20 xyl-5 mtl-1 leuB6 thi-1*)	Promega
BL21 pLysS	An *E. coli* strain which is lysogenic for λ-DE3 and contains the T7 bacteriophage gene I, encoding T7 RNA polymerase under the control of the *lac* UV5 promoter as well as a plasmid, pLysS, which carries the gene encoding T7 lysozyme (Cm ^r^)	Promega
MC1061	*E. coli* hsdR mcrB araD139 Δ (araABC-leu)7679 ΔlacX74 galU galK rpsL thi	[76]
**Plasmids**		
pSUP202	pMB1 replicon (Cb^r^, Tc^r^, Cm^r^)	[77]
pSUP202-Rck	Vector carrying the *rck* gene (Cb^r^, Cm^r^)	[14]
pSUP202-PagN	Vector carrying the *pagN* gene (Cb^r^, Cm^r^)	This study
pSUP202-Inv GFP	Vector carrying the *invasin* gene from *Yersinia enterocolitica* and *gfp* gene (Cb^r^)	[14]
pGEX-4 T-2	Fusion vector carrying the glutathione S-transferase gene (Cb^r^)	GE Healthcare
pGEX-4 T-2114–159 Rck	Vector carrying the glutathione S-transferase (GST) gene linked to 113–159 *rck* gene (Cb^r^)	[10]
pGEX-4 T-2 PagN	Vector carrying the glutathione S-transferase (GST) gene linked to *pagN* gene (Cb^r^)	This study
pcDNA 3.1 Wp85	Vector carrying the wild-type bovine *p85α* sequence (Cb^r^)	[12]
pcDNA 3.1 ∆p85	Vector carrying the mutant bovine p85α sequence (Cb^r^)	[12]

*Cb*^*r*^ carbenicillin resistance, *Tc*^*r*^ tetracyclin resistance, *Cm*^*r*^ chloramphenicol resistance

### Expression of Wp85α and mutant ∆p85α in CHO cells

CHO cells stably overexpressing Wp85α or ∆p85α were obtained as described by Mijouin et al. [12]. Selection was started by adding G-418 at 1 mg/ml to the cell culture medium. For pcDNA3.1 and Rev. pcDNA3.1 primers (listed in Table 3) were used to screen by polymerase chain reaction (PCR) the resistant CHO cells expressing each protein. Proliferation of Wp85α and mutant ∆p85α CHO cells was similar as described previously [12].

**Table 3:** Primers used in this study

Primer name	Sequence (5′ to 3′)
*pagN EcoRI* forw	CTC GAA TTC ATT AAG GCA GGT TCT GAA ATG
*pagN* NcoI rev	TCT CCA TGG TTA AAA GGC GTA AGT AAT GCC
*pagN-*GST forw	CTC GGA TCC CAT CAT CAT CAT CAT CAT AAA GAA GGG ATC TAT ATC ACC GGG A
*pagN*-GST rev	TCT GAA TTC TTA AAA GGC GTA AGT AAT GCC GAG
Forw pcDNA3.1	GAC TCA CTA TAG GGA GAC CCA AGC TGG CTA
Rev pcDNA3.1	GCT GGG CAA CTA GAA GGC ACA GTC GAG GCT

### DNA constructs

The *pagN* gene was amplified from wild-type *S.* Typhimurium 14028 strain by PCR (polymerase chain reaction) using *pagN* EcoRI forw primer (flanked by EcoRI restriction site) and *pagN* NcoI rev primer (flanked by NcoI restriction site) and cloned into pSUP202 expression vector [77], before being transformed into *E. coli* HB101. The same method was used to construct the (His)_6_-*pagN* without its signal peptide (PagN*-*GST) into pGEX-4 T-2 expression vector (Amersham-Pharmacia), using primers *pagN-*GST forw and *pagN*-GST rev, flanked by BamHI and EcoRI restriction sites, before being transformed into *E. coli* BL21 pLysS. In Table 3, primer sequences used in this study are listed.

### Adhesion and invasion assays

Cells were cultured in 24-well tissue culture plates (Falcon) to obtain a confluent monolayer. They were infected for 60 min at 37 °C with bacteria in DMEM without FBS.

For adhesion assays, after infection, cells were washed at least four times with PBS (phosphate buffer saline, Sigma) and then lysed at 4 °C with distilled water. Viable bacteria (extra- and intra-cellular) were counted after plating serial dilutions on TSA (Tryptic Soy Agar).

The number of internalized bacteria was determined using a gentamicin protection assay to kill extracellular bacteria, as previously described [11]. After 90 min treatment with gentamicin at 100 μg/ml (Gibco), cells were washed and lysed in cold distilled water. The number of internalized bacteria was enumerated as before [11].

### Flow cytometry

The CHO, pgsA745, HT29 and Caco2 cells were fixed for 15 min in PFA 2% (paraformaldehyde) at 4 °C and then washed with cold wash buffer containing BSA at 0.5% (bovine serum albumin). Cell samples were saturated with PBS containing BSA 2.5% at 4 °C for 15 min. The mouse anti-heparin/heparan sulfate (HS; clone T320.11, Millipore) was diluted to 1:40 in PBS containing BSA 1% and incubated with cells for 45 min on ice and then washed three times. As secondary antibody, Alexa 488-conjugated goat anti–mouse antibodies (Invitrogen) diluted to 1:200 in PBS containing BSA 1% were used and incubated with cells for 45 min on ice. After three washes, cells were resuspended in PFA 2% and then the relative fluorescence of the cell lines was analyzed using a LSR-Fortessa X-20 analyzer (BD Biosciences). The relative surface expression of HS on cells is expressed as the percentage of positive cells (determined by Overton subtraction of isotype control histograms from labelled histograms [78]).

### Expression and purification of recombinant protein

Recombinant GST-tagged PagN and 114–159 Rck proteins were induced in *E. coli* BL21 pLysS transformed with pGEX4T2-PagN or pGEX4T2–114-159 Rck upon treatment with IPTG 1 mM (isopropyl β-D-1-thiogalactopyranoside, Sigma) for 4 h as previously described [10]. For protein purification, cells were harvested by centrifugation, resuspended in buffer containing Tris pH 8 50 mM, EDTA 40 mM, sucrose 25%, MgCl_2_ 100 mM, Triton X-100 0.2%, PMSF (phenylmethylsulfonylfluoride) 1 mM and cOmplete Protease Inhibitor Cocktail (Boehringer) and sonicated. After clearing, fusion proteins were affinity-purified from the soluble fraction on Glutathione-Sepharose 4B beads (Amersham Biosciences) following the manufacturer’s instructions [10].

### Coating of latex beads

2 μm diameter latex beads (polystyrene sulphate modified, Sigma) were washed and resuspended in PBS containg purified GST-114-159 Rck, GST-PagN and GST proteins. Proteins were adsorbed onto the beads at room temperature for 3 h. After adding BSA (20 mg/ml), the beads were incubated for a further hour at room temperature. The beads were then washed in PBS.

### Immunofluorescence microscopy

Jeg-3 and CHO cells on coverslips were infected with either GST-114-159 Rck-, GST-PagN- or GST- coated beads at MOI 50:1. After incubation for 30 min, cells were washed in PBS to remove unbound extracellular beads. In brief, after fixation of the monolayers in PFA 4%, and permeabilization in triton 0.2%, actin was stained with Rhodamin-Phalloidin (diluted 1:200; Sigma;). Finally, coverslips were mounted in fluorescence mounting medium (Dako) and analyzed with a Leica SP8 confocal laser-scanning microscope (Leica TCS SP8, Germany).

### Scanning Electron microscopy

CHO and Jeg3 cells were grown on coverslips and infected with beads or bacteria to a cell ratio of 100:1. After 30 min of bacteria- or beads- cell contact at 37 °C, cells were washed in PBS and fixed in a mixture of PFA 4% and glutaraldehyde 1% (0.3 M pH 7.4) for 1 h. Samples were then treated for scanning electron microscopy analysis as described in Burlaud-Gaillart et al. [79]. The observations were performed using a Zeiss Ultra plus FEG-SEM scanning electron microscope (Oberkochen, Germany).

### Statistical analysis

Data were analyzed using an unpaired t test or a Mann Whitney test using Prism (version 6.0; GraphPad Software, La Jolla, CA, USA).

## Data Availability

The datasets used to produce the results in Fig. 1 are publically available in Enterobase (https://enterobase.warwick.ac.uk/species/index/senterica). The analysis is available from Olivier Grépinet (olivier.grepinet@inrae.fr).

## Abbreviations

AHL: N-acyl-L homoserine lactones
BSA: Bovine serum albumine
Cb: Carbenicillin
Cm: Chloramphenicol
DKO: Integrin β1 double knockout
DMEM: Dulbecco’s modified Eagle’s medium
DMSO: Dimethyl sulfoxide
EGFR: Epidermal growth factor receptor
FBS: Fetal bovine serum
GST: Glutathione S-Transferase
Hd: Haplotype diversity
HS: Heparan sulphates
HSPG: Heparan sulfate proteoglycan
IPTG: Isopropyl β-D-1-thiogalactopyranoside
LB: Luria-Bertani
MOI: Multiplicity of infection
PAF: Paraformaldehyde
PagN: *phoP*-activated gene
PBS: Phosphate buffer saline
PCR: Polymerase chain reaction
PI 3-kinase: Phosphatidylinositol 3-kinase
PMSF: Phenylmethylsulfonylfluoride
Rck: Resistance to complement killing
R: Richness
SCV: *Salmonella*-containing vacuole
SPI-1: *Salmonella* pathogenicity island-1
Tc: Tetracyclin
TKO: Integrin β1 triple knockout
T3SS-1: Type III secretion system-1
TSA: Tryptic soy agar
